# Porous Organic Materials in Tissue Engineering: Recent Advances and Applications for Severed Facial Nerve Injury Repair

**DOI:** 10.3390/molecules29030566

**Published:** 2024-01-23

**Authors:** Jingxuan Sun, Wenxin Cao, Shuang Pan, Lina He, Dongchao Ji, Nannan Zheng, Xiangyu Sun, Ranxu Wang, Yumei Niu

**Affiliations:** 1 The First Affiliated Hospital of Harbin Medical University, School of Stomatology, Harbin Medical University, Harbin 150001, China; 2018021032@hrbmu.edu.cn (J.S.); panshuang@hrbmu.edu.cn (S.P.); helina426@163.com (L.H.); sxy20080316@126.com (X.S.); 2 National Key Laboratory of Science and Technology on Advanced Composites in Special Environments, Harbin Institute of Technology, Harbin 150080, China; caowenxin@hit.edu.cn (W.C.); dongchaoji@163.com (D.J.); 3 Zhengzhou Research Institute, Harbin Institute of Technology, Zhengzhou 450000, China; 4 Key Laboratory of Micro-Systems and Micro-Structures Manufacturing (Ministry of Education), School of Medicine and Health, Harbin Institute of Technology, Harbin 150001, China; zhengnanll@126.com

**Keywords:** porous organic materials, facial nerve injury, facial nerve regeneration, tissue engineering, nerve conduit

## Abstract

The prevalence of facial nerve injury is substantial, and the restoration of its structure and function remains a significant challenge. Autologous nerve transplantation is a common treatment for severed facial nerve injury; however, it has great limitations. Therefore, there is an urgent need for clinical repair methods that can rival it. Tissue engineering nerve conduits are usually composed of scaffolds, cells and neurofactors. Tissue engineering is regarded as a promising method for facial nerve regeneration. Among different factors, the porous nerve conduit made of organic materials, which has high porosity and biocompatibility, plays an indispensable role. This review introduces facial nerve injury and the existing treatment methods and discusses the necessity of the application of porous nerve conduit. We focus on the application of porous organic polymer materials from production technology and material classification and summarize the necessity and research progress of these in repairing severed facial nerve injury, which is relatively rare in the existing articles. This review provides a theoretical basis for further research into and clinical interventions on facial nerve injury and has certain guiding significance for the development of new materials.

## 1. Introduction

Facial nerve injury, caused by tumor, trauma and other factors, often leads to facial sensory and functional aberrations, including facial expression muscle paralysis, loss of taste, etc., seriously affecting the patients’ quality of life [1]. For clinicians, regaining the structure and functionality of the severed facial nerve is still a difficult undertaking [2]. The key to restoring the structure and function of the facial nerve is to use its own repair capacity and surgical operations to reconnect nerves and muscles. Despite significant advancements in facial nerve injury repair resulting from extensive research and technological innovation, the efficacy of existing repair methods remains limited by various factors such as the cause, location, and severity of injury, as well as the age and overall health of patients [3,4,5]. Potential therapeutic options comparable to autologous nerve transplantation are the key to facial nerve healing.

The development of biological alternatives for tissue or organ healing via tissue engineering, which merges cell biology and material science, offers fresh hope for the treatment of facial nerve injury. Cells, growth factors, and biological scaffolds are the main elements of tissue engineering. Among them, the design and manufacture of appropriate scaffolds is one of the key factors. This is because the scaffold is in direct contact with the cell or tissue surface and can provide structural support and direction for the regeneration process. The ideal scaffolds can simulate the microenvironment of tissues and provide structures for cell communication, tissue growth and nutrient delivery. In order to achieve these goals, researchers have begun to explore effective construction methods for biological scaffolds.

Neural guidance scaffolds with various structures have been studied. Among them, porous scaffolds have been gradually applied to the preparation of tissue engineering scaffolds due to their good porosity, large specific surface area, strong operability, and good mechanical properties [6]. These characteristics can enhance the adherence of seed cells, the exchange of materials at the application site, the loading rate of signal components, and the effectiveness of signal transmission. Additionally, porous materials with a certain morphological structure (directional or multi-channel structure) can exhibit anisotropy and resemble the extracellular matrix in terms of their structure (ECM: a complex network of macromolecules, whose complex network structure can provide material and structural support for the location of injury and provide a place for enhancing the biological behavior of cells through the transmission of signaling molecules), which can influence both the biological activities of cells and the recovery process of nerve tissue [7]. During this period, the choice of scaffold materials is crucial because they are the fundamental parts that come into contact with cells or tissues directly and influence the physical and chemical properties of scaffolds. Numerous organic compounds, including both natural and artificial polymers, have been investigated or applied [8,9].

The application of a porous nerve conduit with an organic polymer as raw material in facial nerve regeneration is the focus of this review. We reviewed the application history of nerve conduits in facial nerve injury in recent decades and summarized the application and development of tissue engineering technology in facial nerve injury repair. We discussed the clinical application value of porous nerve conduits and also analyzed the potential for accelerating the clinical implementation and technological advancement of artificial nerve conduits. This will provide the possibility of and theoretical basis for the organic integration of material science and tissue engineering with biomedicine in the future.

## 2. Facial Nerve Injury and Repair

**Figure 1:**
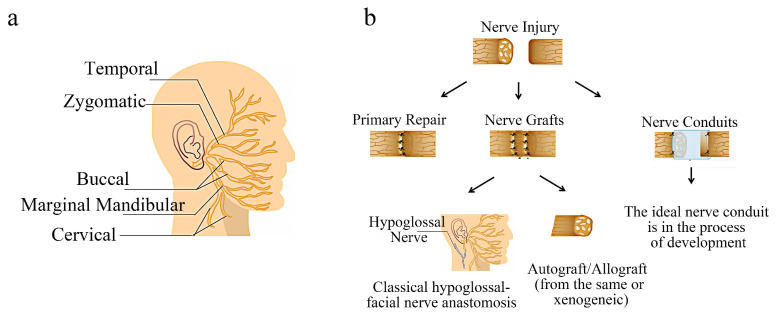
Facial nerve injury and repair: (**a**) schematic representation of the facial nerve anatomy. (**b**) current common treatment methods of facial nerve injury. The concept of diagram is derived from open-source research.

The facial nerve, also known as the seventh cranial nerve, is indeed a mixed nerve that primarily consists of motor fibers and is responsible for controlling the muscles of facial expression, parasympathetic regulation including the secretion of sublingual glands and submandibular glands, and taste sensation in the anterior 2/3 of the tongue [10]. The facial nerve has a special anatomical structure and superficial position (Figure 1a). It is easily damaged due to trauma and tumor, which seriously affect the physical and mental health of patients [11]. Especially in the field of oral and maxillofacial surgery, some radical surgery needs to sacrifice the facial nerve to achieve the purpose of the treatment [12]. Although the facial nerve has a certain self-repair ability, it is very limited; therefore, effective methods of nerve repair are necessary.

### Current Common Treatment Methods of Facial Nerve Injury

Several nerve healing methods started to be used in the field of face nerve injury treatment in the very late nineteenth and early twentieth centuries (Figure 1b), including direct suture, nerve transplantation, neuromuscular pedicle transplantation, microvascular muscle transfer, etc. [13]. At present, the common repair methods in clinics are direct suture and nerve transplantation [14]. The effect of direct repair is better, but it is suitable for a case of a small gap in the injury site without tension in the suture [15]. However, in clinical practice, such cases are rare. If the end-to-end suture is forced in order to simplify the surgical process and achieve the purpose of direct suture, complications such as nerve ischemia and scar formation will occur, resulting in dysfunction [16].

When there is a nerve gap, nerve transplantation is often required, including autologous and allogeneic nerve transplantation (from the same subject or via xenogeneic transplantation), among which autologous nerve transplantation is a better choice. Typically, the sural nerve or the great auricular nerve, which can offer long-distance donors, are chosen as the source of donors for nerve transplants [17]. When the nerve stumps close to the brainstem cannot be used due to large defects or excessive depth in the temporal bone, other motor nerves are often used to restore facial nerve function in clinical practice, including contralateral nerve transfer techniques, hypoglossal–facial nerve repair techniques, and masseter nerve transfer techniques [18,19,20], but the above repair methods still have certain limitations. For instance, nerve transplantation frequently necessitates the sacrifice of healthy nerves and the loss of nerve function in the donor location. Meanwhile, the functional recovery impact following transplantation will also depend on the degree of matching between the donor and recipient. In addition, the means of nerve transfer will also be limited by the distance of the nerve defect. When the distance is large, there will be greater tension, affecting the repair of nerve function [21].

Nerve transplantation or nerve transfer technology often require the sacrifice of healthy nerves. At the same time, they put forward higher requirements for the skills of the surgeon and the psychological and physiological ability of the patients because they often require longer operation time and higher treatment costs and may cause some complications. Therefore, it is necessary to find an artificial nerve conduit with good performance for the effective repair of facial nerve injury.

## 3. Tissue Engineering Nerve Conduits

The nerve conduit joins the nerve stumps, allowing for the process of nerve restoration with a structural foundation. As the nerve regeneration chamber of the severed nerve, it not only mimics the Bungner zone structure in the physiological process to guide the regeneration of nerve tissue, but also provides a space for axon regeneration to prevent fibrous tissue from growing into the defect space and interfering with the nerve repair process. It also provides a good regeneration microenvironment for the injured site in terms of promoting the repair of the injury. At present, the competition in the global nerve conduit market is fierce, and a variety of nerve conduit products are being rushed to market. However, the repair effect still needs to be improved [22]. The shortage of nerve graft donors and the large clinical demand make the development of nerve graft materials urgent.

### 3.1. Requirements of the Tissue Engineering Nerve Conduits

There is no surface morphology for the original nerve conduit, nor a dense and impermeable structure that inhibits nerve regeneration [23]. With the deepening of research, it has been found that an ideal neural scaffold can provide a three-dimensional (3D) growth space and repair biophysical environment for cells and tissues. Additionally, different topological structures play a positive role in the process of nerve repair and the biological behavior of seed cells, including proliferation, migration, and differentiation [24]. Although many researchers have optimized the materials, preparation methods and structures of nerve conduits, excellent nerve conduits suitable for clinical applications are still under study. An ideal facial nerve conduit needs the following elements [25]: (1) good biocompatibility: good biocompatibility of scaffold materials can reduce host immune rejection, provide a better microenvironment for repair of injury, and reduce the generation of scarring at the injury site; (2) suitable biomechanical properties: appropriate mechanical properties can maintain the spatial basis required for regeneration to accommodate facial expression muscle movements, surgical sutures and traction; (3) appropriate porosity and selective permeability: porous materials have a large surface area and rough surface structure, which is conducive to cell adhesion and drug or nutrient factor loading. At the same time, the selective permeability of materials can provide sufficient nutrient and oxygen supply to the injured site and prevent the loss of cells, ensuring the necessary material exchange and the active biological behavior of seed cells [26]; (4) topology: nerve conduits with finer and more directional surface or internal structure help to improve the microenvironment at the injury site and prevent nerve dislocation and scarring. Directional topology facilitates the oriented growth and extension of cells and regenerated tissue, which is very important for neural tissue regeneration [27,28,29]; (5) beneficial and sustainable biological activities: the purpose of tissue engineering is to regenerate tissue, repair damage, or rebuild structure and function, requiring some basic active ingredients, that is, seed cells or signaling molecules. Effective proliferation and differentiation of seed cells, cell–cell interactions, and stimulation and regulation of biological molecules provide great help for the process of facial nerve regeneration [30,31]. In addition, the conductivity [32], plasticity and degradability of the conduits should also be considered.

At present, some nerve conduits endorsed by the Food and Drug Administration, like Axoguard^®^ and Neuragen^®^, have also achieved some clinical effects [33], but the effect of restoration needs to be improved. Moreover, with the progress of research, we found that the advantages of a simple material are limited. By combining different materials, advantages are integrated and synergy is achieved. It is beneficial to improve the quality of conduits. For example, the introduction of nanomaterials like graphene into scaffolds can promote the electrical conductivity of scaffolds, which is beneficial for the differentiation of nerve cells and the repair and regeneration of nerve tissues [34,35]. The incorporation of natural and artificial materials can enhance scaffold mechanical properties and prolong the degradation time. Therefore, it is necessary to develop nerve scaffolds with outstanding performance to provide a better structural basis and regeneration environment for nerve regeneration in order to accelerate nerve repair and regeneration.

### 3.2. Development and Prospect of Tissue Engineering Nerve Conduit

For a new bioengineering technology to be developed to meet clinical needs, preclinical studies are necessary. The animal models for preclinical studies of facial nerve injury repair include rodents, rabbits, and miniature pigs [36,37], among which rodents and rabbits are widely used because of their relatively low operation difficulty. The facial nerve distribution of these experimental animals is similar to that of humans.

Ryo Sasaki and Hajime Matsumine (Tokyo Women’s University, Japan) have made certain contributions to the field of preclinical experimental animal facial nerve anatomy and long-distance injury models [38,39,40]; meanwhile, they have made some progress in the field of facial nerve repair. They transplanted type I collagen gel containing seed cells as a silicone tube filling material into a 7 mm gap in the buccal branch of the Lewis rat facial nerve. They discovered that the graft could repair the electrophysiological function of the facial nerve lesion in rats, and the repair effect was comparable to that of autologous nerve transplantation. However, the silicone tube often required a second operation and caused some rejection of the host. Therefore, researchers began to look for biodegradable nerve conduit materials to apply them in the repair of facial nerve damage. Around this period, Sasaki et al. showed that cell-containing collagen fiber hydrogel loaded with degradable poly (lactic-co-glycolic acid) (PLGA) and polyglycolic acid (PGA) could promote facial nerve regeneration. At the same time, in the process of research, they found that tissue engineering nerve conduits loaded with seed cells or trophic factors played a positive role during nerve regeneration [41,42,43,44,45,46,47,48].

From the initial dense non-degradable hollow conduit to the nerve regeneration chamber with selective permeability and a varied surface morphology, including the functional nerve conduit loaded with nerve factors and seed cells, considerable advancements have been achieved in the development of tissue engineering facial nerve conduits. The advantage of tissue engineering scaffolds is that they can provide good structural support and a strong microenvironment for the repair process, which is the core of the whole neural tissue engineering repair process. To give full play to its advantages, the design of the nerve conduit structure and the selection of materials are very important. They are important components that directly come into contact with cells and tissues and guide nerve regeneration, which determine the progress and development direction of tissue engineering nerve conduit-related fields [49,50].

## 4. Porous Organic Materials in Facial Nerve Injury

**Figure 2:**
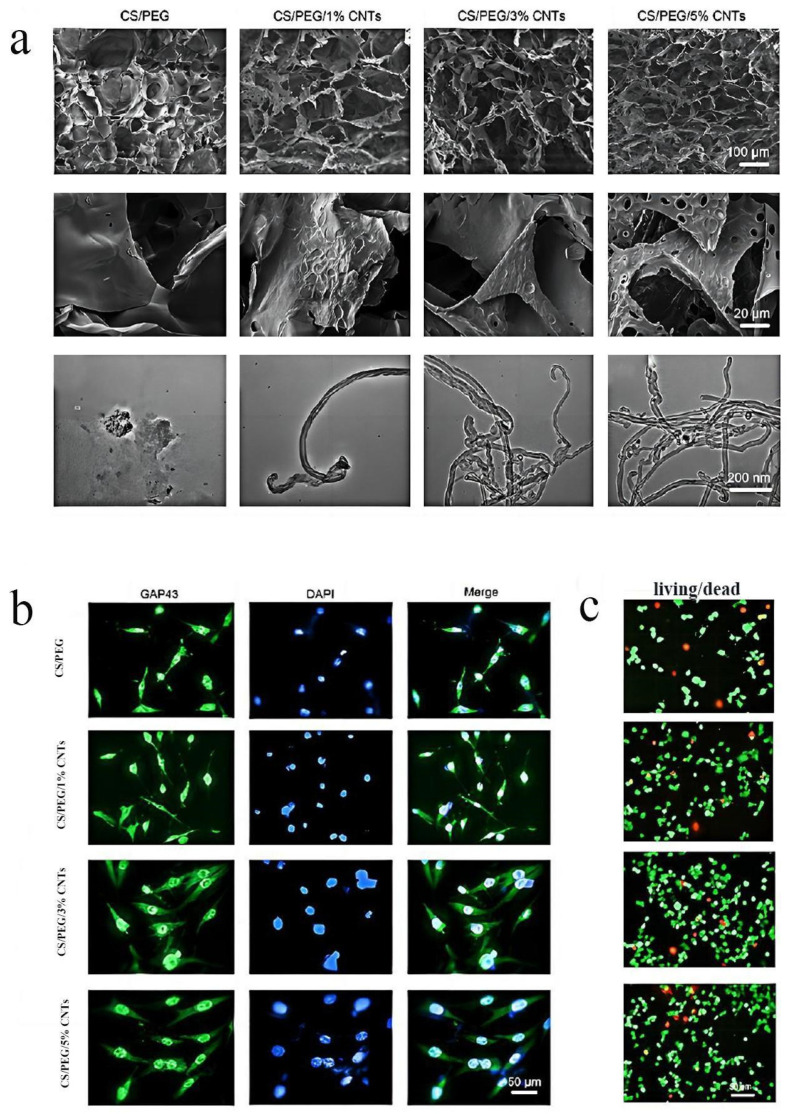
The interconnected porous structure of organic materials promotes the growth and differentiation of cells [34]: (**a**) SEM and TEM images of the lyophilized scaffolds surfaces. For CNT concentration, 1% = 0.01 g/mL. (**b**,**c**) Immunofluorescent identification of cells grown on CS/PEG/CNT (among which, the concentration of CNT differs from 0, 1, 3 to 5), the numbers of living (green) and dead (red) cells on 3d (**c**), and the expression of GAP43 protein (The green fluorescence signal intensity represents the degree of cell differentiation, DAPI was used to locate the nuclei) on 4d (**b**) were analyzed. SEM and TEM represent scanning electron microscopy and transmission electron microscopy, respectively; CS, PEG and CNT, respectively, stand for: chitosan, polyethylene glycol and carbon nanotube. Copyright © Zhejiang University and Springer-Verlag GmbH Germany, part of Springer Nature 2021. PMC Open Access Subset [Internet]. Bethesda (MD): National Library of Medicine. 2003. Available from https://www.ncbi.nlm.nih.gov/pmc/tools/openftlist/ (accessed on 12 June 2023).

As mentioned above, original nerve conduits like silicon tubes could not undergo material exchange, which had great limitations. A 3D porous structure, with high specific surface area and porosity, is a good protein carrier and has good permeability, which is conducive to the interchange of substances around the injured site [51]. It has been reported that multi-walled carbon nanotubes (MWCNTs) were introduced to optimize the characteristics of chitosan (CS)/polyethylene glycol (PEG) composite scaffolds. Additionally, synthesizing composite conduits with highly connected porous structure via the freeze-drying method could heighten the proliferation, adhesion ability, and expression of neural cell markers of PC12 cells (Figure 2). This may be due to the porous structure increasing the surface roughness of the material, and the interconnected porous structure enhancing the information transmission between cells and promoting the growth and differentiation of cells [34].

**Figure 3:**
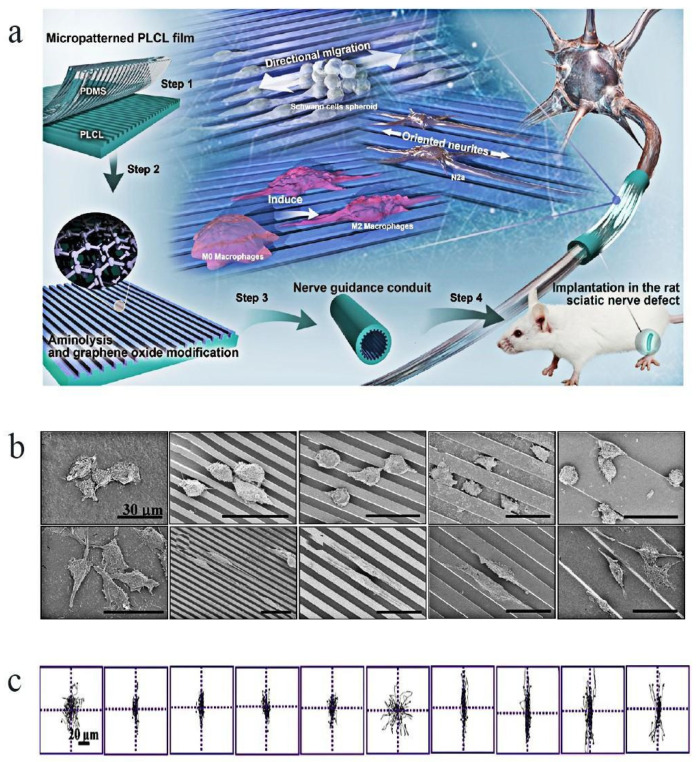
Effect of topology on cells and neural tissues: (**a**) The fabrication step of PLCL film with linear patterns and GO nanosheets and the use of this in vitro and in vivo [29]. (**b**,**c**) Migration of SCs on the fabricated films: (**b**) SEM images of SCs after seeded for 12 h. (**c**) Their migration traces on films with different microfeatures after being seeded for 12 h; the figure showed that the SCs migrated along the strip’s direction [29]. Reprinted (adapted) with permission from Ref. [29]. Copyright 2020, American Chemical Society.

In addition, based on permeability, oriented fiber or groove-like structure can induce the directional growth of cells [24,35] and promote cell differentiation and neurite growth. This helps to preserve the microenvironment in the injury area and avoid the nerve dislocation and scar formation, which is beneficial for the function recovery of nerves. The application of the two topologies alone or in combination achieves surprising results. A composite nerve conduit was prepared by Zhang et al. and they discovered that the NGCs performed better in promoting nerve regeneration. In their investigation, poly(d,l-lactide-co-caprolactone) (PLCL) films were treated with surface aminolysis and the authors performed electrostatic adsorption of graphene oxide (GO) nanosheets to produce linear micropatterns with ridges and grooves of 3/3, 5/5, 10/10, and 30/30 μm. According to Figure 3a–c, the GO-modified micropatterns may greatly speed up the migration of Schwann cells (SCs), and the authors of this work think that this particular subtype of NGC is of great promise in nerve regeneration [29].

**Figure 4:**
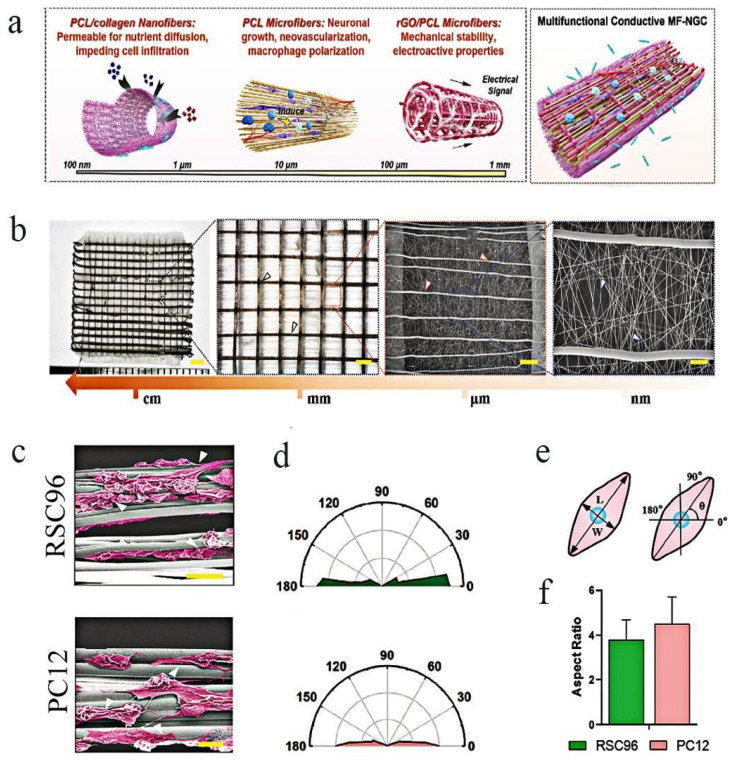
Effect of topology on cells and neural tissues: (**a**) Concept map of a multifunctional conductive MF-NGC for PNI regeneration; the oriented hierarchical microfibers were essential in neurite extension. (**b**) Morphological characterization of the multiscale fibrous scaffold (White, blue and red arrows indicate the PCL/collagen, PCL, and the rGO/PCL microfibers, respectively); (**c**) RSC96 and PC12 cells on NGCs could be seen via SEM imaging on day 7. White arrows represent that neurite grow along with the direction of anisotropic microfibers. (**e**) A schematic representation of the cells’ direction and ratio of aspect of cells. (**f**) Contrast between the aspect ratios of RSC96 and PC12 cells on the NGC scaffold. (**d**) Nightingale rose plots of RSC96 (up) and PC12 cells’ (down) orientation angle on the scaffold [52]. Copyright © 2023 The Authors. Advanced Science published by Wiley-VCH GmbH.

By combining melt electrowriting and electrospinning techniques, Fang et al. created electrical multiscale filled NGCs (MF-NGCs). These NGCs consisted of reduced graphene oxide/PCL microfibers as the core, which can provide the ideal mechanical properties and electrical conductivity to support regenerative processes; random electrospun polycaprolactone (PCL)/collagen nanofibers as the cover, which can offer exceptional penetration for nutrient diffusion and waste removal while preventing the infiltration of fibrous scar tissues; and PCL microfibers as the internal structure, which can provide anisotropic guidance for neuronal growth. Furthermore, in vivo animal studies demonstrated that MF-NGCs can significantly enhance neuronal regrowth and myelination, and also facilitate the functional regeneration of the injured site, as depicted in Figure 4a–f [52].

More and more studies have proved that the porous structure can give nerve conduits better biological characteristics, which is conducive to material exchange, nutrient transport, and cell migration at the injured site. At the same time, it can increase the loading rate of active factors, proteins and cells, which can provide a good repair environment for the injured site, thus promoting the repair of nerve injury [53,54]. Nowadays, with the development of 3D printing and electrospinning technology, the process of preparing porous or some special forms of scaffolds is becoming more and more mature, and research on the application of neural scaffolds has also made great progress.

**Figure 5:**
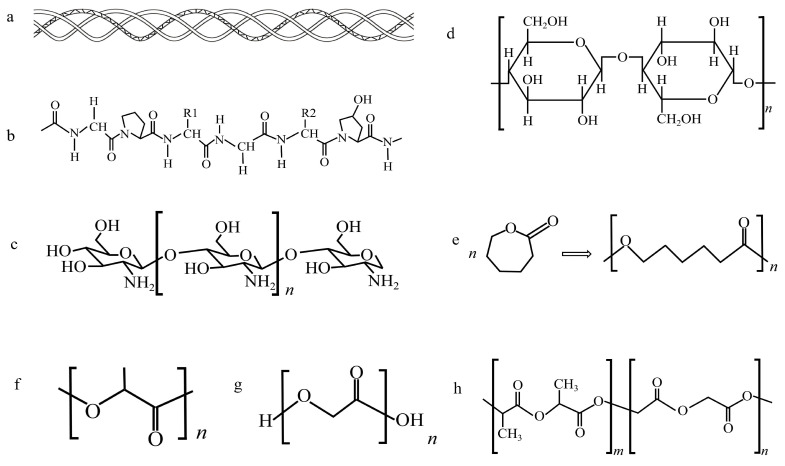
Molecular structure of some common organic polymer materials: (**a**) diagram of the triple helix structure of collagen; (**b**) amino acid sequence of collagen ǀ, which is widely used in the biomedical field; (**c**) chitosan; (**d**) bacterial cellulose; (**e**) bacterial cellulose; (**f**) PLA; (**g**) PGA; (**h**) PLGA. The concept of a structure diagram is derived from open-source research.

Material choice is important during the fabrication process of neural conduits in tissue engineering. The nerve conduits originally used in animals or humans were made of non-degradable synthetic polymeric materials such as silicone, which have made an important contribution to the development of nerve conduits and the early clinical repair of short-distance nerve defects. However, they are not absorbed by the human body and cannot penetrate macromolecules; moreover, their long-term retention in the body will cause chronic inflammation, promote scar formation, and affect the functional recovery of the injured site [38]. With the progress of research, a variety of organic porous materials have been tried to solve this problem, including natural and synthetic materials. Common natural organic porous materials include chitosan, etc., and synthetic organic porous materials include PCL, PLA and PGA, etc., which have attracted wide attention (Figure 5).

### 4.1. Natural Porous Organic Materials

Numerous biomaterials have been tried for nerve regeneration; among them, natural porous biomaterials have the potential to promote cell adhesion, migration, growth, and proliferation. Additionally, because of their high biocompatibility, they can help to avoid harmful effects that are likely caused by inert synthetic materials.

#### 4.1.1. Collagen

Collagen is a kind of structural protein which is a common ECM-based component. The special triple helix structure of collagen gives it excellent properties. Collagen has low immunogenicity, good biocompatibility, and safe degradation products, being easy to fabricate [55,56]. Collagen can be processed into a variety of forms of scaffold materials. Collagen has been widely studied and reported since it was first used as a facial nerve conduit in 1998 [57,58]. Now, researchers have synthesized biodegradable and bioactive collagen nerve grafts [59] to sustainably release neural factors. When used to repair facial nerve defects, collagen conduit can offer a safe regeneration environment and repair space for nerve regeneration. At the same time, when combined with active ingredients or fillings with linear structure, collagen conduits provide a better guidance and repair environment for nerve injury sites, which can more effectively promote nerve regeneration [60]. However, the mechanical properties of collagen are poor, and it is difficult to meet the mechanical requirements of tissue engineering conduits, especially for long-distance defects of facial nerves. Therefore, the mechanical properties of collagen are often enhanced via crosslinking modification or blending with other materials [61,62]. In addition to being used as conduits, some researchers have used collagen as conduit filling material in order to prepare hydrogel sponge or fiber hydrogel, with the characteristics of reticular porous structure helping to load cells and nutritional factors. Additionally, collagen can achieve good facial nerve repair effect [25,63,64,65].

#### 4.1.2. Chitosan

**Figure 6:**
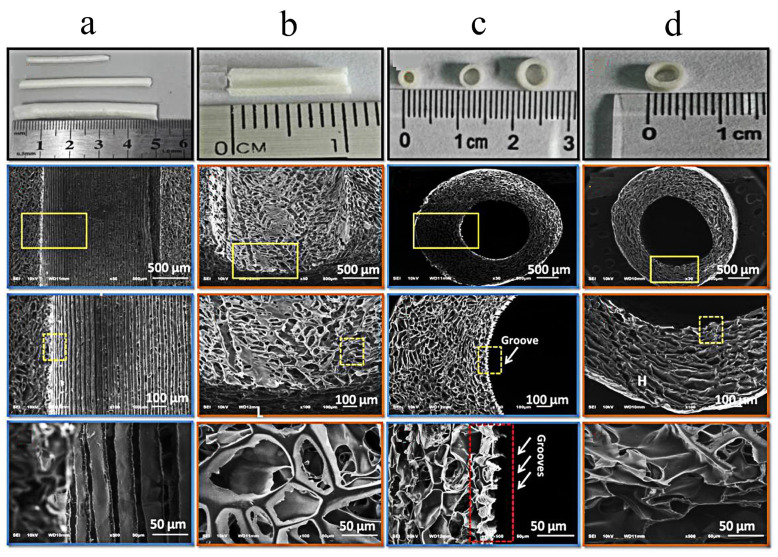

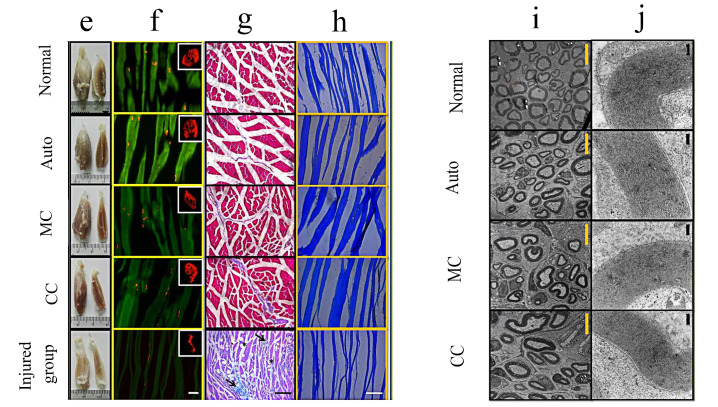
The porous micropatterned chitosan conduits with micropatterned inwalls enhance the 10 mm gapped sciatic nerve regeneration of rats [70]. The prepared chitosan conduits with or without ridge/groove structure on inwall; longitudinal and transverse section of MCs at varying magnifications (**a**,**c**). (**b**,**d**) Image of longitudinal and transverse sections of CCs at different magnifications.(The magnification of yellow rectangles in the figure are listed in the next row, respectively. The red rectangle in (**c**) represents the groove structure on the inside wall of the micropatterned chitosan conduits.) Muscle characterization of (**e**) gastrocnemius (**up**) and anterior tibial (**down**) muscles at the site of operation on rats. Motor endplates in transverse sections of gastrocnemius muscles of operation side in different experimental groups after 12 weeks of surgery (**f**–**h**) represent the immunofluorescence staining Masson trichrome and TBO staining, respectively (Arrowheads and asterisks indicate collagen and angiogenesis, respectively). (**i**,**j**) TEM observation of transverse sections in different groups as mentioned above after 12 weeks of surgery (MC: micropatterned chitosan conduits; CC: chitosan conduits without micropatterned controlled conduits). Reprinted/adapted with permission from Ref. [70]. Copyright 2018, Elsevier.

Chitosan is a natural polysaccharide with a positive surface charge. It is easily absorbed and broken down by the body and has good biocompatibility. Studies have shown that chitosan gel has the effect of promoting facial nerve healing [66]. Meanwhile, it is a good scaffold material for facial nerve tissue engineering [67,68]. The intricate double helix structure of the chitosan molecular chain, which has a lot of hydroxyl and amine groups, makes it simple to create a range of intra- and intermolecular hydrogen bonds and subsequently form derivatives with various purposes. Using cross-linking agents, the molecule’s amine and hydroxyl groups readily cross-link to form a network polymer [69], and its porosity, degradation and selective permeability have great application potential in nerve repair. The porous nerve scaffolds, made of chitosan as the basic material, can significantly promote the repair of nerve tissue. Li et al. used freeze-drying and micromolding technology to develop a porous chitosan micropatterned conduit (MC) with a directed groove in the inner wall (Figure 6a–d). The MC can greatly aid in the regeneration of the damaged sciatic nerve in rats. In this study, MCs with highly arranged microstructure (ridge/groove structure on inwall) can effectively promote the recovery and regeneration of muscles and nerves at the injured site (Figure 6e–h). TEM further confirmed that the MCs can promote the regeneration of peripheral nerves (Figure 6i,j). They suggested that this may be due to the good materialological properties of the MCs. At the same time, the directional formation of new tissue can be accelerated by the directional groove structure, and the recovery of nerve function may be aided by the porous side wall’s ability to load biological substances and lessen nutrient leakage or neurite outgrowth. A breakthrough peripheral nerve regeneration artificial implant can be designed and developed using the experimental and practical foundation that [70] offers. The excellent characteristics of chitosan are making it increasingly widely used in the field of biomedicine. However, there is still not much research on porous chitosan nerve conduits for injury to nerves, and so further research is needed to see how well they work to repair facial nerve damage.

#### 4.1.3. Bacterial Cellulose (BC)

Mostly produced by the genera Acetobacter, Sarcina, and Agrobacterium, BC is a naturally occurring polysaccharide with β-(1,4) glycosidic linkages. It has a reticular fiber structure and can be easily molded and bent into desired shapes without losing its biochemical structure [71,72]. Many studies have utilized BC as a surface coating for wound healing and dural repair because of its benefits. Furthermore, because of its extremely compatible physical and chemical qualities, BC has been employed as a tissue engineering scaffold in organ or tissue restoration. The important and essential role of BC-based biomaterials in neural tissue regeneration has been recognized [73]. For facial nerve injury, the researchers used BC synthesized by Acetobacter xylinum to wrap the nerve stumps and form a nerve guide tube. The short follow-up period may have contributed to the lack of significant differences in whisker movement and electrophysiological test results between the experimental group with a BC tube and the other groups at 10 weeks post operation. However, the use of BC tubes as nerve conduits resulted in a significant increase in the number of regenerating myelinated fibers, and the regenerated nerve with BC tube combined with primary suture had a more regular myelin sheath and no fibrosis. According to this study, BC tubes may facilitate nerve regeneration by creating a conduit for nerve regeneration and shielding the affected location from the surrounding tissue’s deleterious effects on nerve regeneration [74]. Combining the excellent properties of BC with primary suture or other bioactive substances for application to the repair of facial nerve injury has great research potential and is worthy of further exploration.

Natural polymeric biomaterials such as collagen, chitosan and BC have been put to extensive use in tissue engineering nerve repair research (Table 1). These natural polymers are usually biocompatible and can be degraded in vivo. In addition to being used as nerve conduits, they also play a variety of roles in tissue engineering, such as carriers of drugs, cells or active factors [75,76], or as a filler in the conduits [77,78,79]. The regeneration rate of human peripheral nerve is about 1 mm per day, which is slightly faster in rats. In order to meet the structural support required by the regeneration process, the degradation rate of nerve conduit needs to be slower than the recovery rate of injury so as to provide sufficient growth space [80]. However, natural polymers often struggle to meet the mechanical properties and degradation rates required for nerve regeneration, thus limiting their application as nerve conduits alone. Therefore, the application of synthetic polymer materials is gradually becoming widespread.

### 4.2. Synthetic Porous Organic Materials

In addition to natural polymers, another class of promising biomaterials for creating neural conduits is synthetic polymers, whose chemical and physical properties can be tailored. Much progress has not been made in synthetic polymer materials in terms of mechanical properties, but they are often hydrophobic materials, which can affect cell adhesion and cause inflammation or immune rejection more easily [89]. Therefore, it is necessary to modify them or cross-link them with other materials with good biocompatibility to form functional composites to enhance cytocompatibility [90].

#### 4.2.1. PCL

**Figure 7:**
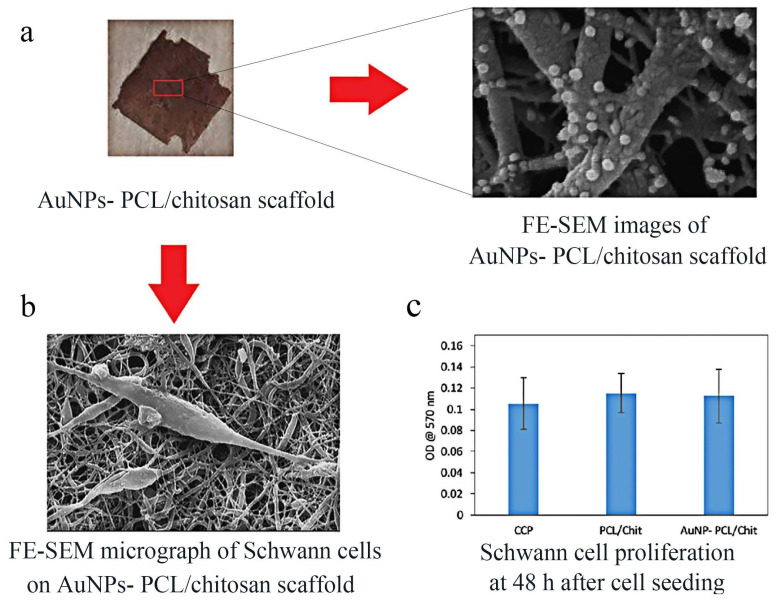
The elongated process of SCs cells with a spindle-shaped morphology (**b**) is supported by the porous conductive AuNP-decorated PCL/chitosan scaffolds (**a**), which exhibit no cytotoxic effect (**c**) [92]. Copyright © The Author(s) 2021.

PCL is a kind of polymer that is formed via ring-opening polymerization of ε-caprolactone monomer with a catalyst present. It is a biocompatible and biodegradable substance that can be utilized as a growth support for cells. As early as 20 years ago, a 10 mm facial nerve defect of a rat’s buccal branch was connected via 3D porous PCL nerve conduits uniformly seeded with Schwann cells and neurotrophic factors [91]. Histological and morphometric analyses were carried out after 4 weeks, and PCL conduits were found to promote facial nerve regeneration. However, the technology was limited at that time, and the restoration effect was inferior to autologous nerve transplantation. The structure and components of the conduit still needed to be further improved. In recent years, PCL nanofibers have been widely utilized in tissue engineering, which can be prepared via electrospinning technology. Some researchers cross-linked PCL nanofibers with collagen and loaded them with active factors to obtain bioactive porous nanofiber scaffolds. These had good cell compatibility and promoted the recovery of facial nerve function [81]. Pooshidani et al. [92] used PCL and chitosan to create a conductive scaffold with regulated porosity. Different percentages of polyethylene oxide (PEO) were used as sacrificial fiber in order to achieve the desired porosity. The results of SEM and MTT showed that the scaffolds with porous structure were successfully synthesized (Figure 7). The potential explanation for the observed phenomenon lies in the fact that the porous fiber structure of the scaffolds offers an enhanced microenvironment and nutrient supply for the regeneration process. This assertion is supported by the viability test and morphological evaluations of the stem cells, which indicate that the hydrophilicity of the material increases with the increase of porosity. Therefore, this improvement in hydrophilicity facilitates cell adhesion, spreading, and proliferation, all of which are crucial factors in the restoration of nerve function.

#### 4.2.2. PLA

PLA, a kind of polyester polymer obtained via polymerization of lactic acid as the main raw material, is a new biodegradable and environmentally friendly material [93]. Its excellent physical and chemical properties make it a promising candidate for biomedical applications [94]. Matsumine, H. et al. created a non-woven, biodegradable nerve conduit made of PLA. This was heated to 90 °C for 20 min while being wound eight times around a stainless-steel core bar in an experimental melt-blown method [95]. After being taken out of the core bar, the layered fabric was employed as a facial nerve conduit. They evaluated its effect on promoting facial nerve regeneration. The results showed that this nerve conduit with porous structure significantly promoted facial nerve regeneration, and that the effect was better than that of the silicone conduit (the PLA tubes showed the largest amount of myelinated neural fibers in the middle of the regenerated facial nerve (mean ± SD, 5051 ± 2335), with autologous nerves (4233 ± 590) and silicone tubes (1604 ± 148) following. The silicone tube group (4.25 ± 1.60 μm) had a significantly smaller axon diameter than the PLA tube group (5.17 ± 1.69 μm), and there was no significant difference between the autograft (5.53 ± 1.93 μm) and PLA tube groups). The authors posit that this phenomenon can be attributed to the favorable permeability of the conduit facilitated by the porous structure. This permeability is advantageous for the development of blood vessels and facilitates the provision of nourishment for nerve repair, thereby fostering the process of nerve regeneration.

#### 4.2.3. PGA

PGA is a synthetic polymer material with friendly biocompatibility and can be degraded by the human body [82]. It has been widely used in the field of biomedicine [83]. The Food and Drug Administration (FDA, Silver Spring, MD, USA) has permitted the use of porous reticular fiber conduits made of PGA in clinical settings. These conduits’ flexible, corrugated walls can withstand twisting and occlusive forces from the surrounding tissue. Moreover, the scaffolds allow oxygen to enter to support the regeneration process and can be absorbed by the body through hydrolysis. Three months after implantation, the tubes start to degrade and they are reabsorbed six to eight months later. Used in combination with mesenchymal stem cells or deciduous tooth stem cells, it has been applied to facial nerve injury and achieved good functional recovery effects [84,85]. Researchers have combined collagen sponge and a PGA conduit to form a porous nerve regeneration scaffold, which is a collagen sponge-filled, resorbable tube composed of PGA that has been cylindrically braided. By immersing the PGA tube in a collagen solution containing 1% volume/weight in hydrochloric acid, amorphous collagen layers are applied to the tube, which is subsequently filled with collagen exhibiting a micro thin-film structure [96]. The tubes have been successfully restoring function to patients over the last few years and this technique has been used since 2002 [97,98]. Related products have been approved by the Japanese government (Nerbridge (Toyobo Co., Ltd., Osaka, Japan)) and are commercially available for practical use in Japan now [43]. This porous composite nerve conduit makes it easier for blood vessels to enter the injured area, thus providing a favorable environment for the regeneration of rat facial nerves [43,44]. Tsujimoto et al. fashioned absorbable PGA fibers into a tube form through knitting, then applied 20 layers of 1% collagen to the tube’s exterior and allowed it to dry. At −80 °C, the lumen was freeze-dried after being filled with 1% collagen. In addition, a 24 h vacuum dehydration heat treatment was performed at 140 °C. Subsequently, researchers established the sympathetic ganglion block (SGB) in beagle. Both functional and morphological assessments revealed that the PGA–collagen tube nerve reconstruction promoted facial nerve regeneration in this model, specifically increasing motor nerve conduction velocity by approximately two times (*p* = 0.018) [99].

#### 4.2.4. PLGA

**Figure 8:**
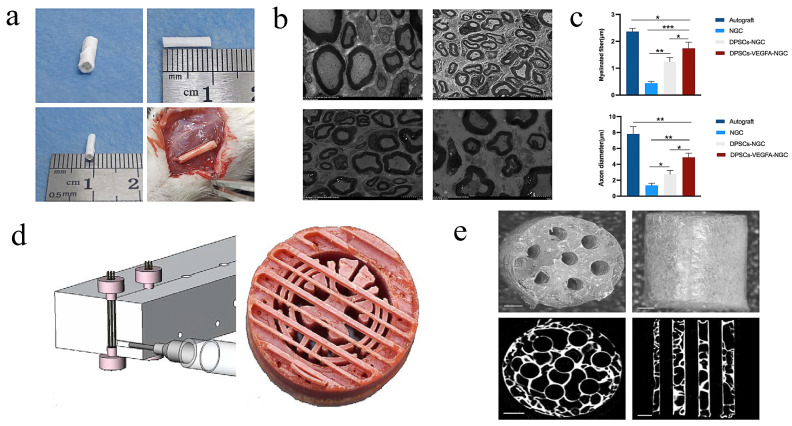
Preparation and application of PLGA conduits: LC-YE-PLGA NGCs combined with VEGFA-modified rDPSCs can speed up the healing process after facial nerve injury in rats [86]; (**a**) pattern of LC-YE-PLGA NGC and its application in facial nerve defects in rat; (**b**) TEM image of axonal and myelin staining in different groups in an order as follows: Autograft, NGC, DPSCs-NGCand DPSCs-VEGFA-NGC group; and (**c**) examination of axon diameter and myelin sheath thickness in each group. * *p* < 0.05, ** *p* < 0.01, *** *p* < 0.001. Data are expressed as the mean ± SD. Copyright © 2023 The Authors’ preparation and morphology of multiple channels and multi-channel scaffolds [102]; (**d**) injection molds used to create multiple-channel scaffolds (**left**) and a sectional view of the parallel-wire injection molding machine used to fabricate multiple-channel scaffolds. A photograph of a wax mold made with PatternMaster that was used to create a scaffold using PLGA injection (**right**); (**e**) parallel-wire molds used to create PLGA scaffolds. (**Top**): photographs, (**bottom**): X-ray microcomputed tomography. (**Left**): cross-sectional view, and (**right**): longitudinal view. Copyright © 2005 Elsevier Ltd.

PLGA is a linear copolymer that can be prepared with varying ratios of its constituent monomers, glycolic acid (GA) and lactic (LA), and end products with different physicochemical properties can be obtained using different preparation parameters [100]. As an important biodegradable biomedical polymer, tissue engineering, regeneration, and controlled medication release are three areas where PLGA is extensively utilized [101]. Sasaki et al. implanted collagen-containing dental pulp cells into degradable PLGA conduits in order to avoid the negative effect of silicone conduits during the repair process. Five days after the procedure, regenerate nerves rebuilt with nerve stumps were observed in 10/10 tubes loaded with dental pulp cells in contrast to only 5/10 control tubes. Nerve stumps in the tubes with dental pulp cells were more effective than those in the control tubes [42]. However, the PLGA conduits used in this study had dense surfaces and no selective permeability, which seemed to be detrimental to the construction of the wound microenvironment. In fact, as early as 2006, Oh et al. used PLGA as raw material to prepare a nerve conduit with a porous structure with selective permeability and hydrophilic qualities via an improved immersion precipitation method. By adjusting the diameter and immersion time of the alginate saline gel rod, the diameter and wall thickness of the tubes could be easily regulated. An asymmetric nanoscale columnar porous structure is seen on the tube wall. With strong mechanical properties and the ability to efficiently penetrate nutrients, this conduit offers great potential for use in maintaining a stable support structure for nerve regeneration [25]. Xu et al. produced dental pulp stem cells overexpressing VEGFA in conjunction with PLGA conduits via electrospinning to heal 10 mm facial nerve defects in rats [86]. The composite conduit availed the growth of axons and myelin sheath (Figure 8a–c), which can hasten the healing process following facial nerve damage and enhance the degree of motor ability recovery. However, the repair effect was still not as good as that of autologous nerve transplantation, and the specific mechanism still needs to be verified. There are still some problems to be solved before its clinical application.

Using injection molding and solvent evaporation, M.J Moore et al. was able to create biodegradable scaffolds with a regulated, parallel-channel structure (Figure 8d). Multi-channel nerve conduits with porous structures (Figure 8e) can be loaded with SCs and they can survive for at least 48 h in vitro. The scaffolds containing SCs can promote the regeneration of axons in vivo [102]. With the continuous development of manufacturing technology, a variety of porous PLGA scaffolds can be fabricated to meet different needs by adjusting the copolymer ratio and other means, and the application will be gradually widened. However, the elucidation of how to achieve greater advantages of materials and their mechanisms also requires us to make greater efforts.

### 4.3. Copolymer or a Blend of Polymers

As research has progressed, scientists have come to understand that it is challenging for a single material to satisfy all of the requirements for the perfect scaffold material. Instead, composite materials—which combine various material types—can combine the best qualities of several materials to make up for the shortcomings of individual materials [103,104,105]. PLGA and chitosan have good biocompatibility and degradability, but a small amount of acid could be produced via the degradation of PLGA, which can cause nerve damage. The metabolites of chitosan are alkaline and can neutralize acidic harmful substances of PLGA.

Xia et al. [87] injected collagen-based 3D scaffolds combined with one of the three components (water, GDNF, and GDNF microcapsule) into the PLGA/chitosan composite nerve conduit to connect the nerve stump. In addition to offering an ideal microenvironment for nerve-guided regeneration, the nerve connector serves as a suitable carrier for the realization of consistent active GDNF microcapsules. Meanwhile, it has a very stable degradation rate and good permeability, which can promote the exchange of nutrients in the surrounding environment. This is vitally important in nerve regeneration, and the likelihood of improper nerve regeneration can be reduced using biodegradable nerve conduits with GDNF microcapsules. In comparison to bridging the nerve conduit with an NTF or by directly injecting GDNF into it, the bridging effect is significantly better.

**Figure 9:**
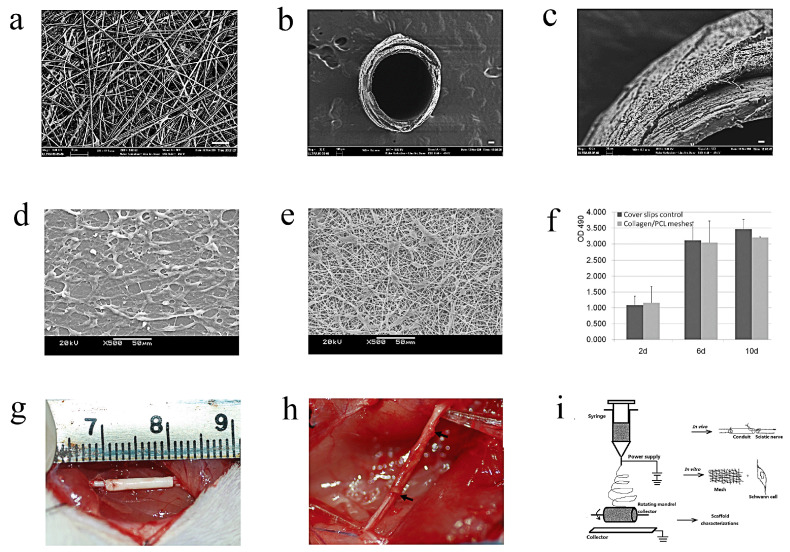
Preparation and application of collagen/PCL porous composite conduits [106]: SEM images of electrospun fibrous membrane and guiding conduits: (**a**–**c**) collagen/PCL fibrous mesh and conduit fabricated via electrospinning and the morphology of collagen/PCL nerve conduit’s outer wall; (**d**–**f**) represent SEM images of the morphology of Schwann cells on different materials: glass cover slips as control group; collagen/PCL fibrous meshes and the proliferation assay after cell seeding via MTS assay on different time points. The difference between the two samples was not statistically significant on days 2, 6 or 10 (*p* > 0.05). In vivo trials of the porous composite conduits: (**g**) rats underwent surgery to surgically implant a collagen/PCL nerve conduit in order to bridge a severed sciatic nerve injury around 8 mm; (**h**) the regenerated nerve was visible four months after surgery. A thin layer of densely packed fibrous connective tissue encircled the conduit. As the regenerated nerve flowed through, the conduit gradually deteriorated, obscuring the initial boundaries (black arrows) between the conduit and nerve. (**i**) Diagram illustrating the three primary components of the experiment as well as the electrospinning apparatus. Electrospun fibers were gathered onto a rotating mandrel and a flat plate collector to create guide conduits and meshes. After testing the electrospun composite mesh in vitro using Schwann cells, two stumps of a rat sciatic nerve lesion were sutured together using guide conduits in vivo. Copyright ©2011 Yu et al.; licensee BioMed Central Ltd.

Yu et al. [106] fabricated PCL/collagen porous composite conduits via electrospinning (Figure 9a,i). Figure 9a of the SEM images displays relatively uniform and bead-free fiber morphologies. On a rotating mandrel with a diameter of 1.2 mm, electrospun collagen/PCL fibers were collected to form a hollow guidance structure, as depicted in Figure 9b,c. The electrospun composite material had better hydrophilic properties (water contact angle changed from electrospun PCL meshes:133.5 ± 0.9 to collagen/PCL fibrous meshes: 0) and elastic properties (Young’s modulus changed from pure PCL: 2.02 ± 0.38 to the composite material: 32.59 ± 1.25). This indicated that, compared to one pure single synthetic polymer, the composite material might be a better substrate for ensuring in vitro and in vivo experimental investigations. Following three days of seeding on the fibrous meshes, SCs were observed via SEM to have adhered and spread well along fibers in in vitro studies (Figure 9d,e). Comparing cells grown on cover slips to those on mesh surfaces, a slightly smaller number of cells were visible (Figure 9e). MTS assay-derived quantitative data on the proliferation of SCs on different surfaces are shown (cell proliferation activity) (Figure 9f). At the aforementioned time points, there are no differences between the different surfaces (*p* > 0.05). In vivo experiments showed that the adult rats’ 8 mm peripheral nerve defects were successfully supported by the porous nerve conduits. The proximal and distal nerve stumps were connected by the regenerated nerves from both the composite conduits and autograft groups. More axons were encouraged to regenerate through the collagen/PCL nerve conduits, and they progressively deteriorated in a way that was consistent with the rate of nerve regeneration (Figure 9g,h). It is thought that this electrospun nerve conduit, with its high cell-attachment surface area and appropriate degradation rate, may be a viable substitute for autograft in peripheral nerve regeneration research. It also holds promise for future use in treating facial nerve regeneration.

Wang et al. [107] designed a novel nerve conduit made of silk fibroin and PLGA copolymer via electrospinning and weaving. The conduit had high porosity, hydrophilicity, tensile stiffness, and good biocompatibility, and is anticipated to offer a dependable scaffold material for the clinical repair of peripheral nerves.

With the progress in material synthesis and bridging techniques, artificial nerve conduits with special structure or surface morphology have been developed. For example, freeze-drying [33,108] can obtain excellent porous structures, gel sponges can provide excellent interconnected porous structures, and polymer electrospinning technology can also obtain nanofibers with porous structures or anisotropy [109]. Moreover, 3D printing can obtain 3D topography with precise topology [110,111,112].

Among these, electrospinning technology can provide fibers with more structure and better performance through the selection of solutions and equipment and via parameter adjustment, such as the orientation, particle size and porosity of fibers, the encapsulation of signal molecules, and the acquisition of a core–shell structure [113]. Additionally, due to the highly bionic characteristics of its products, electrospinning technology has become a high-potential technical means of nerve tissue engineering. However, in terms of product stability and consistency, the use of electrospinning technology to prepare nerve conduits needs further development.

At the same time, 3D printing technology has become a reliable method for preparing biological materials in the biomedical field [114]. Through sophisticated digital design, 3D bioprinting can generate bioimplant porous nerve conduits with controllable morphological and structural properties. Compared with freeze-drying, electrospinning and other methods, 3D bioprinting can generate 3D pore structures with the same porosity and pore size. Zhu [115] et al. developed a rapid 3D printing platform, and the biocompatible neural conduit they produced promoted the tissue and functional regeneration of rat sciatic nerve. At the same time, in order to prove the high usability of their equipment, they also printed bionic tubes that can be anatomically matched to human facial nerves, but there is a lack of relevant research to verify its effectiveness. Three-dimensional printing technology has a high degree of digitalization and good repeatability, but its work efficiency is low and, due to its additive manufacturing characteristics, the precision of the preparation of fine structures, especially the control of the porous size, also needs to be improved [116].

**Figure 10:**
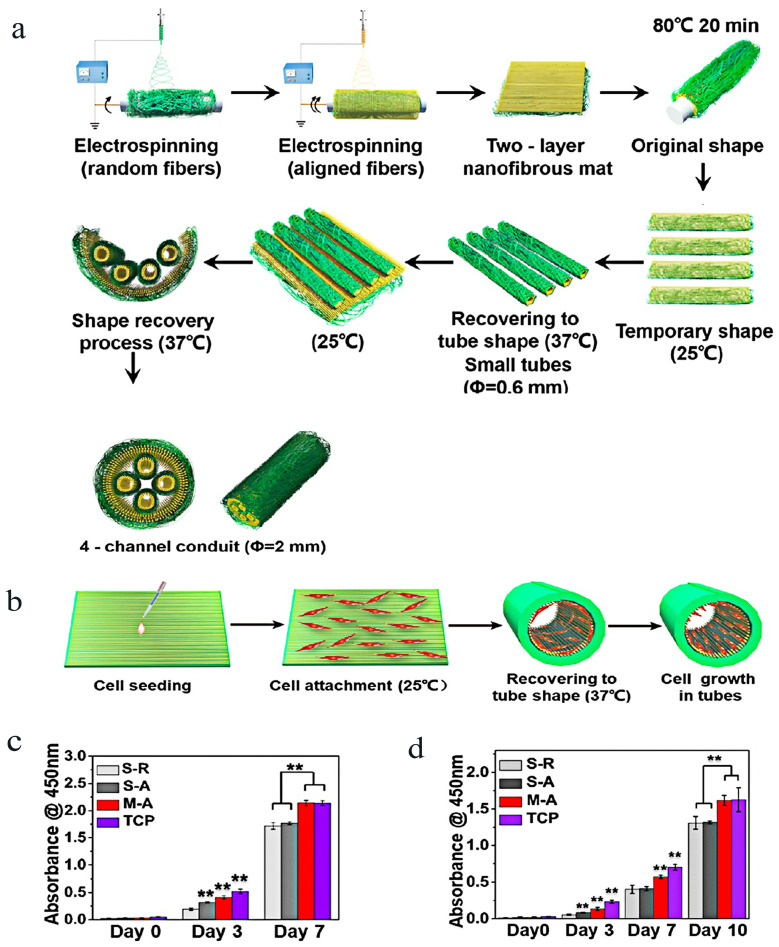
Shape memory nanofiber-based bioinspired multichannel nerve guidance conduit could be fabricated, and this is friendly to the growth of cells [51]: diagram showing how the shape memory nanofibrous mat was used to fabricate the four-channel conduit (**a**) and the process of seeding cells and their growth in various conduits (**b**); (**c**) the proliferation of SCs; and (**d**) the PC12 cells in various conduits. TCP: tissue culture plate. ** *p* < 0.01 means significance (Reprinted/adapted with permission from Ref. [51]. Copyright 2020, American Chemical Society).

**Figure 11:**
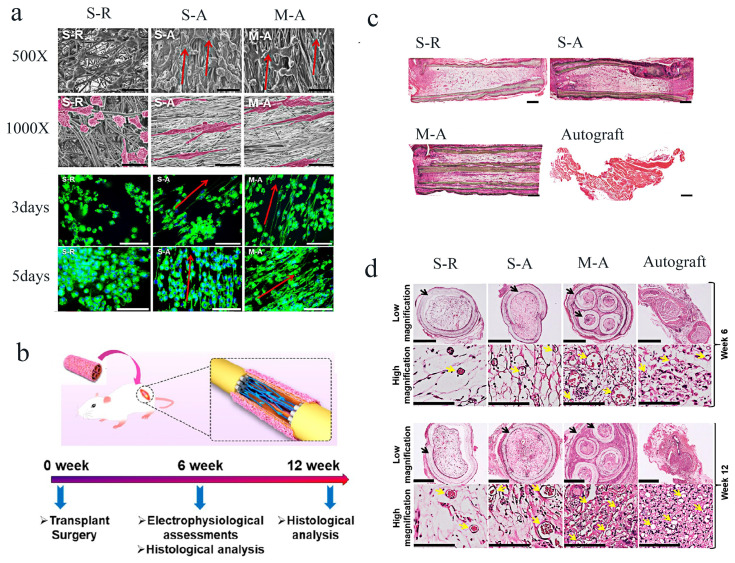
Shape memory nanofiber-based bioinspired multichannel nerve guidance conduit with potential for peripheral nerve repair [51]: (**a**) SCs in different conduits after incubation for several days. Arrows indicate the direction of cells’ growth. (**b**) Diagram showing the implantation step of an M-A conduit into the SD rat sciatic nerve lesion model.(**c**) HE-stained images of the longitudinal sections of the regenerated nerves at 6 weeks following surgery. (**d**) Cross-sections of the center portions of the regenerated nerves were displayed in HE-stained pictures at 6 and 12 weeks following surgery. Yellow arrows mean the fabrication of new blood vessels, and the black indicate the nerve conduits (Reprinted/adapted with permission from Ref. [51]. Copyright 2020, American Chemical Society).

In addition, the selection of some polymers with shape memory function can also realize the fabrication of porous materials or nerve conduits with special morphology [51]. Plasmid PLATMC polymer, which has a degradable shape memory, was used to create a multichannel nerve guiding conduit with geographical cues and self-forming capabilities. The electrospun shape memory nanofibrous mat could be temporarily molded into a planar shape for cell loading, starting with an initial tubular shape produced by a high-temperature molding process, and then obtaining a uniform distribution of cells. After that, it could return spontaneously to its former tubular shape at a physical temperature of around 37 °C, producing a multichannel conduit (Figure 10a,b). Comparing this multichannel-aligned scaffold to the single-channel hollow conduit without filler allowed for greater room for cell development and improved the proliferation of SCs and PC12 cells (Figure 10c,d). In addition, as shown in Figure 11a, the direction of fibers affected the orientation of SCs. When implanted into the body of rats, after several weeks of repair, newly grown nerve tissues extended into all conduits. These newly grown nerve tissues were arranged neatly and clearly along the conduit’s long axis, guided by aligned fibers within the conduits. The multichannel conduit demonstrated this more clearly, confirming that the conduit’s topographic guidance cues were essential in directing nerve regeneration (Figure 11c,d). These findings demonstrate that shape memory polymers can be used to create self-forming nerve conduits. The internal surface of the conduit was made of aligned electrospun nanofibers, which supplied topographical cues for axon elongation and subsequent cell growth, and the conduit performed better in terms of nerve defect repair and cell growth. As a result, there is a lot of promise for peripheral nerve regeneration with the created bioinspired multichannel-aligned nerve guidance conduit.

## 5. Prospects

Facial nerve injury is often caused by trauma, tumors and other reasons, which brings great physiological, psychological and economic pressure to patients, especially the severed facial nerve injury, which has limited self-repair ability, making it difficult to restore nerve function. It is very important to find an effective method to repair the severed facial nerve injury. Tissue engineering, as an emerging discipline that can construct tissues or organs, brings us new ideas. The question of how to follow the biomimetic principle, better simulate the peripheral nerve and the surrounding natural structure, guide nerve regeneration, and prevent nerve tissue dislocation is the focus and challenge of current tissue engineering research.

In recent years, a lot of discussion has focused on the design and material choice for tissue engineering nerve conduits. From the initial dense hollow conduits, nerve conduits with porous structure, groove structure, and multi-channel structure, as well as the use of fibers or hydrogels as fillers, have emerged one after another. Great progress has been made in the design, materials, and synthesis of nerve conduits. For nutrients and growth factors to pass through tissue engineering scaffolds, porosity is a crucial requirement [117]. Appropriate pore size can ensure material metabolism, prevent the loss of active nutrients such as cells, and block the interference of surrounding tissues. Some studies have shown that the ideal pore size is between 5–30 μm, but some have pointed out that peripheral nerve regeneration requires direct contact between cells and axons. Excessively small pores, also called semi-permeable pores (pore size: 1–10 μm), may affect the regeneration process, and some studies have shown that 10–20 microns is an ideal pore size range [118,119]. However, the specific porosity and pore size range have not been reached, which also limits the progress of porous materials in this field to a certain extent. Currently, porous designs are very popular and easy to manufacture. Meanwhile, to prepare NGCs, a variety of polymers—natural, synthetic, and a combination of the two—were employed. However, it is necessary to reinforce the benefits of nerve regeneration and the functional recovery of the regenerated nerve. In the future, the preparation and application of functional NGCs will be the trend of tissue engineering to repair facial nerve injury.

With the deepening of research, we realize that the trophic factors emitted by the injured nerve contribute to the formation of a microenvironment and that the restoration of nerve structure and function is significantly influenced by the microenvironment [120,121]. In the past few decades, tissue engineering nerve conduits have experienced great development. However, improving the nerve conduit’s biochemical, physical and chemical properties and supplying a more suitable microenvironment for injury repair is the goal of an ideal tissue engineering nerve conduit [122]. Nerve conduits are being generated that are biologically functional and can facilitate the repair process. These require some basic active ingredients, that is, seed cells or signaling molecules. The great potential of stem cells has been gradually noticed in the field of neural tissue engineering over the years [29,30]. However, there are no licensed stem cell products. We must investigate further ethical issues of stem cell application, standardized production methods for obtaining stem cells, and the biological basis of stem cell action [123,124]. At the same time, the question of how to efficiently combine NGCs with seed cells and nutritional factors and play a greater role still needs further exploration [106]. The loading methods of cells and factors and the continuous innovation of materials are still in the initial stage of research [62], and great efforts are needed by researchers [125].

During the development process of facial nerve conduits, more preclinical studies were conducted in the experimental animal model of sciatic nerve injury, and there was still a dearth of research on the repair of facial nerve injury [36,126]. The complexity of the facial nerve’s anatomical structure, along with its limited exposure and small operating space, poses challenges in terms of establishing the defect model. The preparation of the conduit and the surgical technique are highly required. Therefore, there is still a large gap in the relevant field. However, the regeneration potential of nerves from different sources and locations is different. Therefore, although some existing studies have achieved satisfactory results in the repair of other peripheral nerves such as sciatic nerve, these results can only be used as a reference for the facial nerve. The development and application of materials and their effect on nerve repair still need to be further explored and verified by researchers in related fields.

In addition, if cell tracing and imaging technology can be better used to show the process of nerve repair in vivo, it will be helpful to explore the fate of seed cells and stimulating factors and the mechanisms of action of tissue engineering nerve conduits. In particular, imaging technology has obvious advantages in the process of visually monitoring peripheral nerve regeneration, and so its combination with tissue engineering nerve conduits has become a hot topic of current research and has achieved certain results [127]. At present, facial NGCs are still in their infancy, and there are many problems to be solved. Simultaneously, the translation of scientific research achievements into clinical applications necessitates preliminary preclinical procedures, including the verification of the effect of long-distance nerve injury repair, the establishment of large-scale experimental animal models and in vivo studies, the formulation of unified standards and evaluation criteria for product production, etc. Researchers in related fields still have a long way to go, but it is reassuring that such work is already underway [88].

## 6. Summary

Facial nerve injury can cause serious physical and psychological damage. Clinical outcomes in these patients remain poor due to limitations in current treatment options and inadequate understandings of the mechanisms of facial nerve injury and repair. In recent years, tissue engineering has made remarkable achievements in the field of regenerative medicine and has great potential to gradually become the core of regenerative medicine. Porosity is an important property of tissue engineering nerve ducts, one which can ensure that the regeneration process is not interfered with by the surrounding tissues and nutrient supply. From the appearance of a dense hollow conduit to the appearance of porous nerve conduit, artificial nerve conduits have made important breakthroughs. In this review, several common materials for porous nerve conduits are summarized, including natural and synthetic polymers and their mixtures, the manufacturing processes of porous nerve conduit and the applications of these in facial nerve injury. We reaffirm the necessity of using porous structures in the design of neural conduits, highlighting the great potential of porous organic materials in the field of tissue engineering. Some suggestions for the future development of tissue engineering nerve conduits are proposed, and we believe that emphasis should be placed on the development of bioactive nerve regeneration chambers with suitable permeability and special surface morphology. This work will provide suggestions for the clinical treatment of facial nerve injury and provide guidance for the future development of tissue engineering nerve conduits that can replace autologous nerve transplantation.

## Figures and Tables

**Table 1:** Application of porous organic nerve conduits in facial nerve injuries.

Materials	Animals	Models	Results	Ref
Collagen	Rats	A 7 mm buccal branch defect	Collagen nerve conduit can effectively repair facial nerve injury, but the repair effect is not as good as that of autologous nerve.	[22]
Collagen	Swine	A 35 mm defect of the buccal branch	The functional COL/nb-TCP nerve conduit combined with NGF and COL filaments could promote facial nerve regeneration.	[29]
Collagen + PGA	Rats	A 10 mm defect in the marginal branch	The nerve function recovery effect of polyglycolate-collagen tube is not as good as that of autologous graft.	[43]
PGA	Wistar rats	A 7 mm buccal branch defect	DFAT cell-filled PGA conduits could promote nerve regenerate process.	[44]
Collagen	Cats	A 5 mm defect in the dorsal ramus	The collagen nerve guide has achieved good facial nerve regeneration effect.	[57]
Collagen	Rabbits	A 10 mm defect in buccal branch	Collagen and E-PTFE composite tubes are effective in the repair of peripheral nerves’ continuity defects.	[58]
Collagen	Rats	A 10 mm defect of the buccal branch	The extent of regeneration (of a large gap) approached that afforded by an autograft in both functional and histological aspects.	[59]
Collagen	Rats	A 4 mm defect in the trunk	The conduit can guide the axons’ orderly growth in the facial nerve transsection model and promote the recovery of nerve function.	[60]
Collagen	Rats	7 mm facial nerve buccal branch defect	ASCs with different differentiation states may have therapeutic potential in facial nerve regeneration.	[61]
Collagen	Rats	A 8 mm defect in ficial buccal branch	The novel artificial nerve conduits formed by immobilizing GDNF in collagen conduits is beneficial to facial nerve repair.	[62]
Collagen	Rats	A 7 mm buccal branch defect	Optimal nerve regeneration was facilitated by the infusion of untreated-SVF into the nerve conduit.	[63]
Collagen	Rats	A 7 mm buccal branch defect	OECs promoted the facial nerve regeneration and the functional recovery of which.	[64]
Collagen	Rats	A 8 mm buccal branch defect	The scaffold effectively promoted the proliferation of NS/PCs in collagen scaffolds due to the sustained presentation of bFGF.	[65]
BC	Rats	A 3 mm defect in the main trunk in facial nerve	BC can be formed into a hollow tube that directs nerve axons, improving nerve regeneration following transection.	[74]
PCL	Rats	A 10 mm defect in the buccal branch	The 3-dimensional cell matrix composed of fibrin/Schwann improved both the amount and quality of peripheral nerve regeneration via PCL conduits.	[79]
PCL	Rats	A 4 mm defect in the main trunk	PCL/CoMF/UCS provides a beneficial environment for facial nerve regeneration.	[81]
PLA	Rats	A 7 mm defect in the buccal branch	Similar results have been obtained with the porous PLA non-woven fabric tube in stimulating peripheral nerve regeneration after autologous nerve transplantation.	[82]
PGA	Wistar rats	A 5 mm mandibular branch defect	In comparison with the autograft group, the regeneration was superior in the group treated with SHED linked to the PGA neurotube.	[83]
PGA	Rats	A 5 mm defect in the mandibular branch of the left facial	Both BMSC and Schwann-like cells within PGAt in rats could enhance facial nerve regeneration, but the Schwann-like cells worked better.	[84]
PGA	Beagle dogs	A 7 mm defect in buccal branch	PGA-c tubes Promote facial nerve regeneration and increase long-term blood flow.	[85]
PLGA	Rats	A 10 mm defect in the buccal branch	VEGFA-treated rDPSCs combined with LC-YE-PLGA NGCs are beneficial to facial nerve regeneration and functional recovery.	[86]
PLGA + Chitosan	Rats	The middle part of the nerve trunk (About 1 mm)	Stable and sustained-release GNDF microcapsules in biodegradable nerve conduits can reduce the dislocation of severed facial nerve.	[87]
Collagen	Minipigs	A 35 mm buccal branch defect	collagen scaffolds containing the neurocytokines bFGF and CNTF have a comparatively better therapeutic effect.	[88]

Abbreviation: bacterial cellulose (BC); polylactic acid (PLA).
